# Absorption of methotrexate under standardized conditions in children with acute lymphoblastic leukaemia.

**DOI:** 10.1038/bjc.1980.286

**Published:** 1980-10

**Authors:** C. R. Pinkerton, S. G. Welshman, S. I. Dempsey, J. M. Bridges, J. F. Glasgow


*Br. J. Cancer* (1980) **42**, 613

### *Short Communication*

### ABSORPTION OF METHOTREXATE UNDER STANDARDIZED CONDITIONS IN CHILDREN WITH ACUTE LYMPHOBLASTIC LEUKAEMIA

C. R. PINKERTON*, S. G. WELSHMAN§, S. I. DEMPSEY†, J. M. BRIDGES*† AND J. F. T. GLASGOW*‡

*From the *Royal Belfast Hospital for Sick Children and the Departments of †Haematology and ‡Child Health of The Queen's University of Belfast, and the §Department of Clinical Chemistry, Belfast City Hospital*

Received 18 June 1980 Accepted 2 July 1980

THE IDENTIFICATION of an individual patient's methotrexate (MTX) absorption characteristics would be of clinical value if this could be used either to anticipate toxicity or to indicate inadequate absorption. The therapeutic range of MTX blood levels is currently unknown. It is not clear whether either response or prognosis depends upon the magnitude of the peak level, the duration for which a critical level is exceeded, or the total amount of drug absorbed as indicated by the area under the absorption curve. It has recently been suggested that the rate of absorption may be an important factor, with the slow absorber having a high relapse rate (Craft, 1979 *Int. Symp. Methotrexate*).

Variations in the absorption of orally administered MTX were first demonstrated by Freeman-Narrod (1962) who suggested that patients could be divided into fast and slow absorbers based upon the serum MTX level 1 h after administration. The slow absorber had a low peak level, but MTX was detectable for a prolonged period, often associated with mucosal ulceration. An association between prolonged MTX therapy and drug malabsorption has been suggested (Freeman-Narrod, 1962; Craft, 1977) since this drug is known to affect small intestinal structure (Trier, 1962) and function (Craft, 1977; Venho, 1976).

Previous studies of MTX absorption have been poorly standardized with regard to the dose of MTX and its relationship to food and other oral medication (Freeman-Narrod, 1962; Kierney, 1979; Boomla, 1979). In this study these factors were carefully standardized, and the early serum profile of MTX was measured in a group of leukaemic children.

Twenty children with acute lymphoblastic leukaemia (ALL) were studied, 10 of whom were males. Their ages ranged from 3 to 16 years and the duration on therapy from 3 to 36 months. All were managed according to the Medical Research Council's Working Party on Leukaemia in Childhood (UKALL trials) and were receiving MTX either as a single weekly dose or as a 5-day course every 3 weeks. No other therapy was given for at least 5 days prior to investigation.

Patients were fasted from 20:00 hours and next morning a fine venous cannula was inserted. A resting blood sample was taken and oral MTX (15 mg/m$^2$) given with 20 ml water. Blood samples were taken at 20-min intervals for the first hour and hourly thereafter up to 5 h. Serum was separated within 6 h of sampling and stored at −20°C. Samples were analysed within 2 weeks by enzyme-linked immunoassay (Emit MTX assay, Silva, Maidenhead); levels less than 0·2 $\mu$M were assayed by

Correspondence to: Dr C. R. Pinkerton, Department of Child Health, Institute of Clinical Science, Grosvenor Road, Belfast BT12 6BJ, Northern Ireland.

Fig. 1.—Mean serum MTX concentration ±s.d. in 20 patients. Conversion of SI to traditional units: 1 μM = 454 ng/ml.

Fig. 2.—Methotrexate absorption patterns in slow absorbers, with one hour levels less than 0·44 μM. Conversion: SI to traditional units—1 μM = 454 ng/ml.

radioimmunoassay using the principle of competitive protein binding ($I^{125}$ MTX-RIA, Uniscience, Cambridge).

Results are shown in Figs 1 and 2. There was wide variation in peak concentrations (0·28–1·6 μM) with a mean of 1·01 μM. The timing of the peak value ranged from 0·5 to 4 h after administration, mean 1·5 h. Fig. 2 shows the absorption patterns of 6 patients (30%) who had serum levels less than 0·44 μM at 1 h and who could be classified as slow absorbers (Craft, 1979). After 1 h there was wide variation in the absorption pattern, 3 patients reaching high peak levels which were greater than the mean for the group as a whole.

It seems clear from these observations that a 1h serum MTX level may not accurately reflect the overall serum profile. In contrast to the observations of Freeman-Narrod (1962) the slow absorber may reach high peak levels indicating adequate absorption, despite a low level at 1 h. At this early stage the serum level may be influenced by several factors, such as the rate of gastric emptying and small intestinal motility, as well as the rates of intravascular distribution and excretion. It may be important to determine which of these factors contribute to an early low level, if in fact the slow absorber is predisposed to early disease relapse (Craft, 1979). A more extended analysis of the absorption/excretion profile would provide information such as the absorption rate constant, elimination rate constant and the area under the curve. It might more logically be these derivations which would be correlated with response to therapy and rate of relapse. Moreover, although this study was carried out in the fasting state, investigation of the influences of differing nutrients taken in association with the drug is being carried out, as these may significantly alter absorption (Krondl, 1970).

On the basis of random sampling and division into fast or slow absorbers it would be unwise at present to alter methotrexate therapy either in terms of dosage or route of administration. Although information can be obtained from short-term studies such as this, it is preferable that data be obtained from a more extended study, to permit accurate pharmacokinetic analysis.

We are grateful to Mr H. Mackey and Mr M. McMaster of the Belfast City Hospital and to the haematology laboratory staff of Royal Belfast Hospital for Sick Children, for technical assistance.

CRP is in receipt of a Royal Belfast Hospital for Sick Children Research Fellowship.

### REFERENCES

Boomla, D., Aherne, G. W., Greaves, M. W. & Quinton, M. (1979) Radioimmunoassayable methotrexate concentrations in plasma and skin exudate of patients with psoriasis. *Clin. Exp. Dermatol.*, **4**, 457.

Craft, A. W., Kay, H. E. M., Lawson, D. N. & McElwain, T. J. (1977) Methotrexate induced malabsorption in children with acute lymphoblastic leukaemia. *Br. Med. J.*, ii, 1511.

Freeman-Narrod, M. (1962) The pharmacology of methotrexate. In: *Methotrexate in the Treatment of Cancer.* Baltimore: Williams & Wilkins. p. 17.

Kierney, P. J., Light, A. P., Preece, A. & Mott, M. G. (1979) Unpredictable serum levels after oral methotrexate in children with acute lymphoblastic leukaemia. *Cancer Chemother. Pharmacol.*, **3**, 117.

Krondl, A. (1970) Present understanding of the interaction of drugs and food during absorption. *Can. Med. Assoc. J.*, **103**, 360.

Trier, J. S. (1962) Morphological alterations induced by methotrexate in the mucosa of human proximal intestine. *Gastroenterology*, **42**, 295.

Venho, V. M. K. (1976) Effect of methotrexate on drug absorption from the rat intestine *in situ* and *in vitro*. *Acta. Pharmacol. Toxicol.*, **38**, 450.


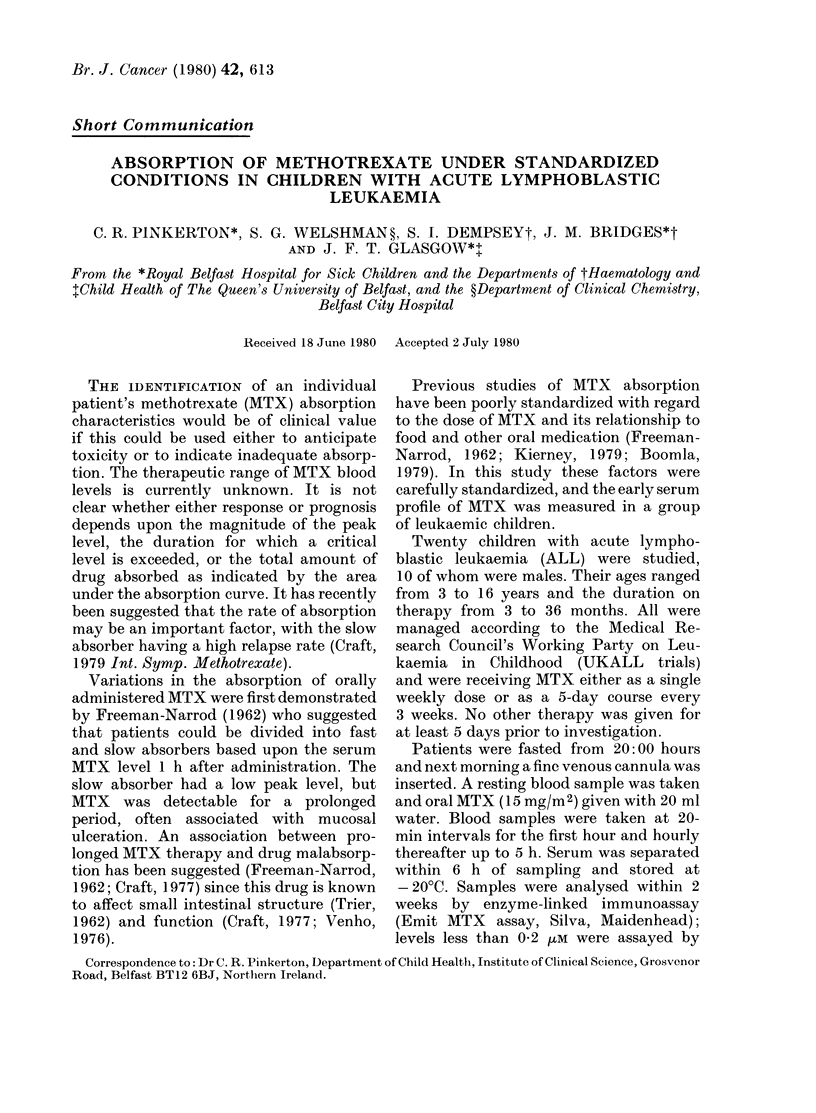

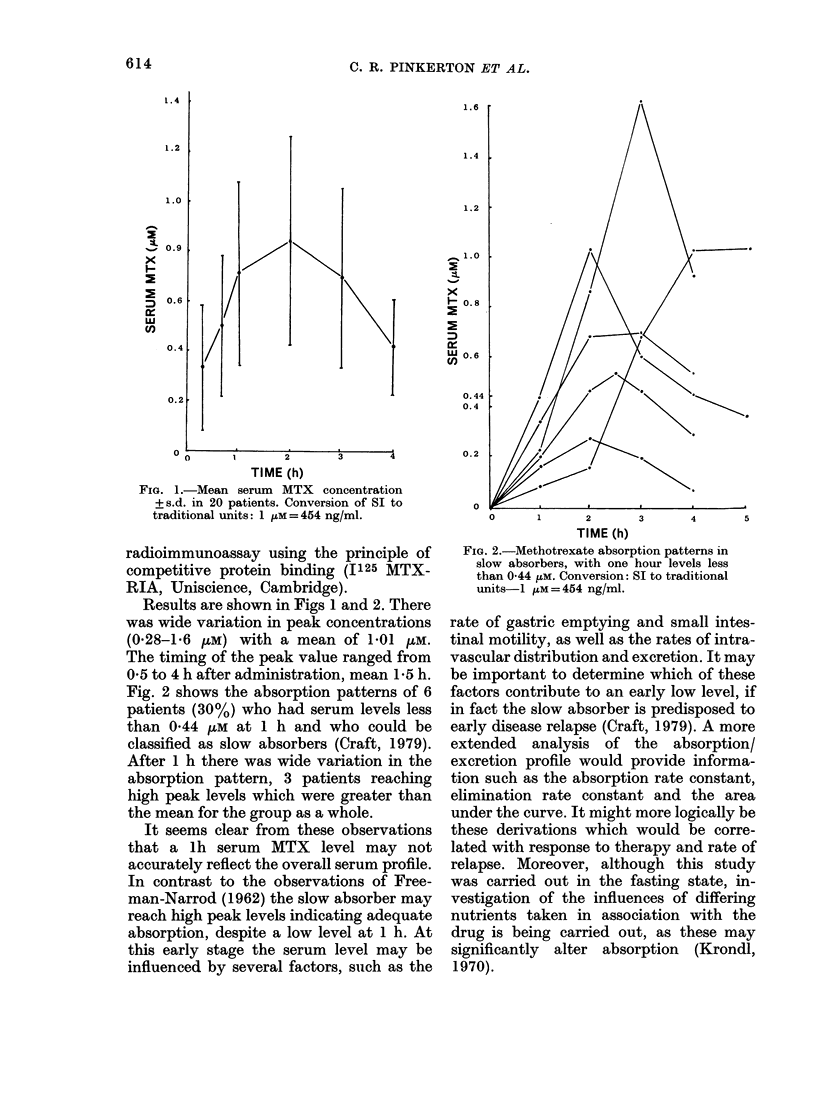

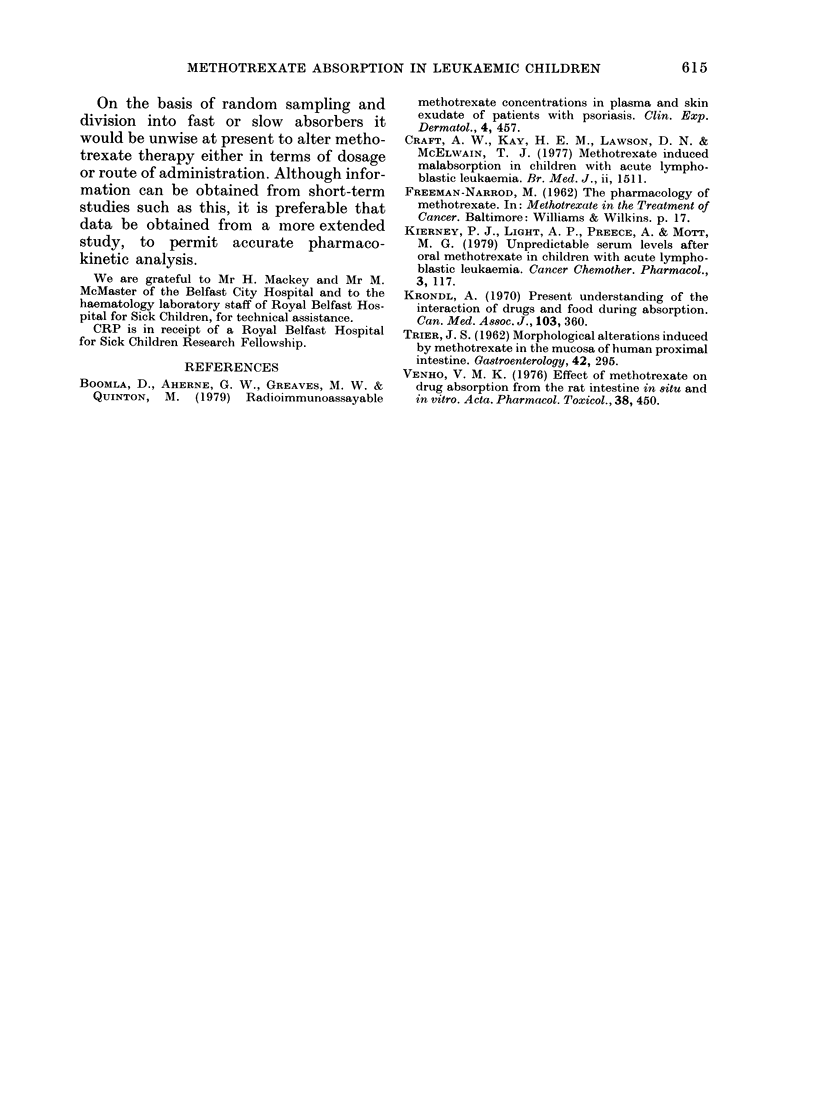


## References

[OCR_00245] Boomla K., Aherne G. W., Greaves M. W., Quinton M. (1979). Radioimmunoassayable methotrexate concentrations in plasma and skin exudate of patients with psoriasis.. Clin Exp Dermatol.

[OCR_00264] Kearney P. J., Light P. A., Preece A., Mott M. G. (1979). Unpredictable serum levels after oral methotrexate in children with acute lymphoblastic leukaemia.. Cancer Chemother Pharmacol.

[OCR_00271] Krondl A. (1970). Present understanding of the interaction of drugs and food during absorption.. Can Med Assoc J.

[OCR_00276] TRIER J. S. (1962). Morphologic alterations induced by methotrexate in the mucosa of human proximal intestine. I. Serial observations by light microscopy.. Gastroenterology.

[OCR_00281] Venho V. M. (1976). Effect of methotrexate on drug absorption from the rat small intestine in situ and in vitro.. Acta Pharmacol Toxicol (Copenh).

